# The Effectiveness of Early Rehabilitation in Limiting the Progression of Idiopathic Scoliosis

**DOI:** 10.3390/jcm13051422

**Published:** 2024-02-29

**Authors:** Marek Kluszczyński, Katarzyna Zaborowska-Sapeta, Ireneusz Kowalski, Ilona Karpiel

**Affiliations:** 1 Collegium Medicum, Jan Dlugosz University, 4/8 Waszyngtona, 42-200 Częstochowa, Poland; 2 Department of Rehabilitation and Orthopedics, School of Medicine, University of Warmia and Mazury in Olsztyn, 10-082 Olsztyn, Poland; 3 Regional Specialized Children’s Hospital in Olsztyn, 10-561 Olsztyn, Poland; 4 Łukasiewicz Research Network—Krakow Institute of Technology, The Centre for Biomedical Engineering, Zakopianska Str. 73, 30-418 Krakow, Poland

**Keywords:** functional leg length discrepancy, scoliosis, pelvis asymmetry, body posture

## Abstract

**Background:** The purpose of this study was to assess specific rehabilitation methods’ effectiveness in early idiopathic scoliosis (IS) development, focusing on lower limb functional inequality’s role in scoliosis progression. **Materials and Methods**: This study comprised 812 patients aged 6–16 years at risk of developing idiopathic scoliosis (IS). The mean (SD) age was 10.66 (3.16) years. Patients were categorized into high- and medium-risk groups based on the angle of trunk rotation (ATR) size. Specific scoliosis physiotherapy was used, and the average follow-up period was 28.1 ± 14.5 months. Changes in ATR, Cobb angle, and functional length of the lower limbs pre- and post-treatment were statistically analyzed across three age groups (6–9, 10–12, and 13–16 years) and three scoliosis locations. **Results**: Significant effectiveness of early rehabilitation was observed in the high-risk group of children aged 6–9 years. In the medium-risk group, significant reductions in ATR were observed in both the 6–9 and 10–12 age groups across all three scoliosis locations. Additionally, there was a significant decrease in the Cobb angle in the thoracolumbar region and a significant reduction in lower limb inequality across all age groups and scoliosis locations. **Conclusions**: The early implementation of specific physiotherapy may enhance the efficacy of idiopathic scoliosis treatment by attenuating factors contributing to its progression.

## 1. Introduction

Idiopathic scoliosis (IS), as a three-dimensional deformity of the spine, manifests in apparently healthy children and can develop during any rapid period of growth depending on numerous factors [1,2,3]. Researchers affiliated with international scientific societies such as the Scoliosis Research Society (SRS) and the Society on Scoliosis Orthopedic and Rehabilitation Treatment (SOSORT) have formulated standardized principles for the conservative treatment of IS, emphasizing early diagnosis and employing physiotherapeutic specific scoliosis exercises (PSSEs), bracing, education, and surgical intervention in cases of conservative treatment failure [4]. The shift towards conservative treatment for IS, as opposed to the previously favored surgical approach, is attributed to the efforts of numerous developers and researchers of conservative methods, whose publications have provided strong evidence supporting the efficacy of PSSEs [4], bracing [2,5,6], and combined therapy [4,7]. This consistent approach and advancements in conservative treatment have led to a twofold reduction in surgical interventions despite a stable incidence of the disease [8]. Early diagnosis is critical for the success of conservative treatment; however, despite significant improvements in screening test quality, approximately 70% of children with IS are still diagnosed too late [9,10]. According to currently accepted SOSORT criteria, children of preschool and school age undergoing periodic posture examinations by school nurses have only a 40–50% chance of being diagnosed with scoliosis at an early stage [11,12].

It has been demonstrated that relying solely on the criterion of the trunk rotation angle (ATR) for predicting the development of back asymmetry may lead to significant errors [12,13,14]. To enhance the diagnosis and effectiveness of conservative treatment for IS, the authors propose considering two aspects in conservative IS treatment based on reports in the field of scoliosis pathomorphology and etiopathogenesis. Firstly, they suggest lowering the ATR threshold for qualifying for PSSEs in younger children aged 6–12 years. Reports indicate that more than half of children with an ATR of 5–6° may have scoliosis with a Cobb’s angle of 15° in the lumbar or thoracolumbar region [13,14]. Burwell and Park referred to this period in a child’s life as the pre-scoliotic period, which typically precedes the sudden onset of scoliosis during puberty by several months and is characterized by slight trunk asymmetry, often accompanied by other postural disorders [15,16]. The second aspect involves considering factors contributing to scoliosis progression (FCSPs), which may manifest early in scoliosis formation and influence its progression [15,16]. Documented FCSPs include pelvic asymmetries and functional leg length discrepancies (FLLDs) [17,18,19,20], impaired sagittal spine balance [21,22,23], peripheral joint hypermobility (HJM) [24,25], and weakened deep sensation and proprioception disorders [26,27], often correlated with HJM and impaired neurological balance control [28,29,30,31].

In this study, the occurrence of FLLDs as a clinical symptom of morphological and/or functional asymmetry of the hip girdle, often associated with IS development, was particularly analyzed [17,18,19,20,32,33,34,35,36,37,38,39,40,41]. Pelvic asymmetry may manifest as an oblique position of the sacrum, clinically evidenced by differences in the level of posterior superior iliac spines (SIPSs), as well as octal pelvic torsion, characterized by differences in SIPS level with oppositely oriented differences in anterior superior iliac spine (ASIS) levels [42,43,44,45]. Therefore, the commonly used method of assessing FLLDs, which involves measuring the distance from the ASIS to the medial malleolus with a tape measure, has demonstrated significant measurement error [46,47,48]. In this study, a modified test according to Cooperstein et al. [49] and Travell et al. [50] was used to assess FLLDs, wherein heel levels were assessed in a child lying in a supine position as recommended by Fryer et al. [51]. The reliability of FLLD assessment in clinical practice was confirmed by 78.8% of SOSORT panelists [4], which is why this test has been utilized in our center for several years to evaluate hip girdle function and monitor IS therapy progress.

These aspects formed the basis for early PSSE utilization in a group of children with ATR limits (5–6°) who exhibited at least one FCSP. This group, termed the medium-risk group for scoliosis (MRS), was identified in this study, while children with ATR ≥ 7° constituted the high-risk group for scoliosis (HRS).

In the study, the implementation of PSSEs in the MRS group, comprising children in the pre-scoliotic period, aimed to achieve, among other objectives, the symmetrization of the iliac girdle. This approach was intended to enhance neural mechanisms involved in motor and postural control, thereby influencing the proper development of the spine [13,14,24,25,26,27,28,29,30,31].

This study aimed to assess the use of PSSEs in the early stages of scoliosis development, taking into account changes in FLLD as a contributing factor to progression.

## 2. Materials and Methods

### 2.1. Study Design

This study was approved by the Jan Dlugosz University Ethical Committee, resolution KE-U/10/2021 of 28 September 2021, and conducted in accordance with the Declaration of Helsinki. All parents of the participants were informed of this study’s purpose and examination process, and they provided written consent before the study was initiated.

A prospective cohort study was conducted using a clinical database encompassing all patients referred to our center between 2008 and 2018. The center is a clinic specializing in the treatment of idiopathic scoliosis.

Data collection occurred during routine clinical practice at the center. Children with suspected scoliosis, posture defects, or other conditions requiring rehabilitation were referred to the center by local physicians, pediatricians, orthopedists, and physiotherapists.

During the initial visit, the attending physician informed the patients and their parents/guardians about the type of examination and therapeutic procedures to be employed, including the potential need for X-ray diagnostics. Written consent was obtained from the parents/guardians for each X-ray examination.

### 2.2. Population

**Figure 1:**
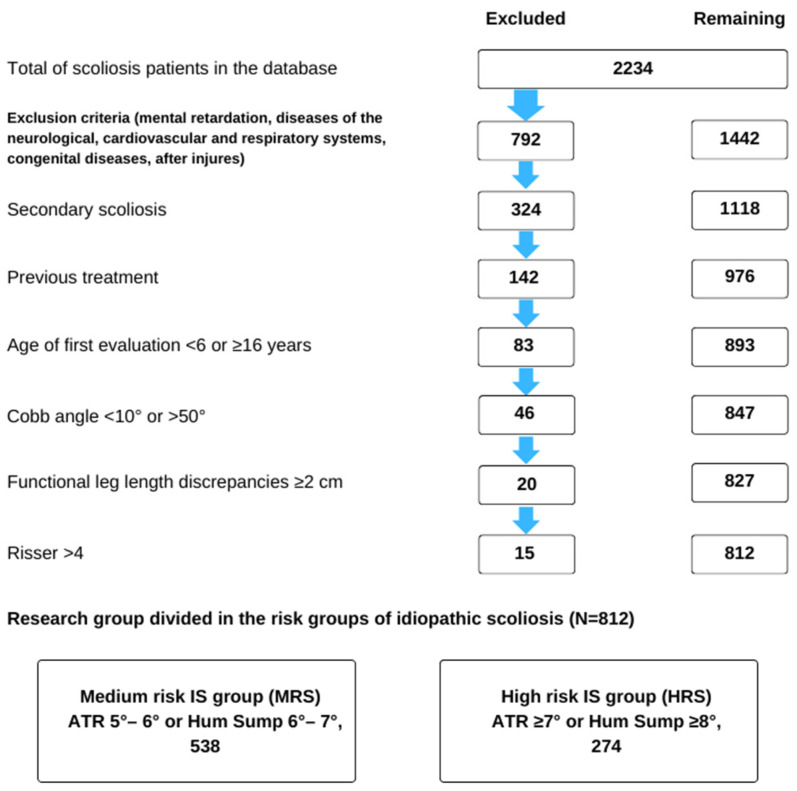
Flow chart of study subject selection from the entire sample of patients included in the clinical database.

The study sample comprised 812 children aged 6–16, including 280 (34.5%) boys and 532 (65.5%) girls, with an average age of 10.66 (SD = 3.16) years. The participants were selected from a group of 2234 individuals treated at the center. The children were categorized into three age groups: 6–9, 10–12, and 13–16 years (Figure 1).

Participants were included in the study based on the following criteria: age; dorsal asymmetry (ATR) ≥ 5° (diagnosed for the first time) or Hump sum ATR ≥ 6°; spinal curvature detected by X-ray with features of idiopathic scoliosis and a Cobb angle ranging from ≥10° to <50°; at least two months of previous scoliosis treatment; and a Risser sign ≤ 4. Figure 1 provides a summary of the exclusion criteria.

The participants were then divided into two groups based on the ATR criterion measured during the initial examination. The medium-risk scoliosis (MRS) group had an ATR ≥ 5° or a Hump sum of 6–7°, and the high-risk scoliosis (HRS) group had an ATR ≥ 7° or a Hump sum ≥ 8°. This division allowed for the observation of changes in group size following treatment.

### 2.3. Methods

**Figure 2:**
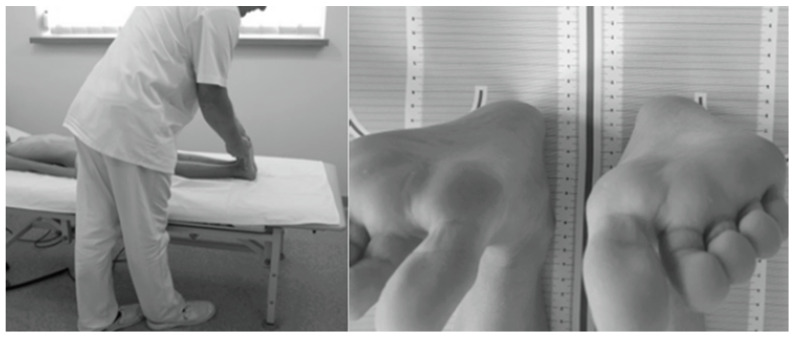
Measurement of FLLD in the HL test in the supine position.

The study protocol involved assessing ATR in the Adams test using a Bunnell scoliometer [44,45] and evaluating the presence of FLLDs according to Cooperstein [49] as modified by Travell et al. [50] and Fryer et al. [51]. The level of the lower surfaces of the heels was assessed with the child lying supine with the lower limbs evenly placed along the trunk, straight at the knee joints, with the ankle joints in intermediate positions between pronation and supination and between dorsiflexion and plantar flexion. The assessment was performed twice to eliminate measurement errors related to uneven adherence of the pelvis to the ground or non-axial positioning of the lower limbs in relation to the torso. In the latter case, the assessment was preceded by a slight pull-up and flexion of the lower hip joints. A heel level difference was diagnosed when the height of each heel differed by ≥0.5 cm (Figure 2) [46].

If the value of the FLLD was ≥2 cm, the absolute length of the lower limbs was additionally measured using a tape to detect structural shortening of the lower limb, which was an exclusion criterion [33,38]. A clinical examination was performed on average every three months, and a spinal X-ray was performed once a year according to SOSORT recommendations, unless otherwise advised by an orthopedic clinician. 

The Cobb angle was measured twice by a single examiner blinded to the patient’s data. The test results were calculated as the average of both measurements. If an ATR ≤ 5° was found during the follow-up visit, no additional X-rays were performed in order to avoid exposing the child to side effects [52].

Based on the clinical examination, the children qualified for early specialized rehabilitation according to the Dobomed method. This method incorporates elements of the Schrott, SEAS, and Lyon methods according to the model developed in the center in consideration of SOSORT recommendations [4].

Each child followed a generalized treatment plan, though not all children performed the same exercises. The selection of exercises was determined primarily by the type of IS, i.e., the number and location of curves, but was also adapted to possible orthopedic deficits (muscle contractures, knee or tarsal valgus) or sensorimotor deficits (posture, balance, and coordination disorders).

Classes were conducted in small groups of 5–6 children by three physiotherapists once or twice a week, with sessions typically lasting for 90 min. Parents participated in the classes, and the children were also advised to perform exercises daily at home for 20 min under parental supervision.

Among the HRS group, 57 patients (21%) were treated with a personalized Cheneau orthosis, according to the SOSORT criteria (Cobb angle of the greatest curvature of scoliosis ≥20°and Risser degree of 0–3) [47]. Despite the recommendation to wear the Cheneau brace 23 h a day, parental feedback indicated that the actual daily usage ranged from 6 to 12 h. The bracing period lasted from six to 38 months. The treatment was conducted by a team of two rehabilitation physicians, eight physiotherapists, and two orthopedic technicians, all in close collaboration.

During therapy, all patients maintained normal physical activity and attended physical education classes at school.

### 2.4. Data Analysis

The parameters examined in this study were evaluated using basic descriptive statistics. Changes in the number of risk groups before and after treatment were compared, and an analysis of patient flow between groups was conducted. Changes in ATR and Cobb angle were determined and further categorized into spinal sections (thoracic, thoracolumbar, and lumbar). For ATR, a change was identified as a result that differed by at least 2 degrees in two consecutive assessments. Percent curve improvement (Cobb angle decrease ≥ 6°), stability (Cobb angle change within ±5° of baseline), and progression (Cobb angle increase ≥ 6°) were compared [4].

The final stage of this study evaluated the changes in FLLDs over the course of therapy, determining the percentage of patients with a shorter right or left limb as well as limb equality before and after the intervention. Additionally, the sums of success percentages (leg alignment) and lack of changes were compared for different spine sections using the proportion test. A significant difference in FLLD of 0.5 cm was assumed for the three locations of asymmetry or scoliosis and used to determine on which side the functional shortening of the limb occurred.

### 2.5. Statistics

The normality test was assessed using quantile–quantile plots (Q-Q plots). To assess changes in patient flow over time between risk groups, a McNemar test was used to analyze dichotomous variables. The nonparametric Wilcoxon signed-rank test was used to compare differences in the Cobb angle and ATR before and after treatment, and the Fisher’s proportion test was used to assess changes in FLLDs. For all analyses, a significance level of 0.05 was used.

## 3. Results

This study included 812 patients, with an average age of approximately 11 years (±3 years), with girls comprising the majority (65.5%). The largest group, representing just over 40% of the participants, consisted of children aged 13 to 17. Approximately one in three individuals were aged 10 to 12, and the remaining group comprised the youngest participants, aged 6 to 9. The MRS group predominated, forming two-thirds of the study population (Table 1).

Approximately one-third of the children underwent X-ray examinations, with 14.7% of patients receiving the examination at least twice. The Cobb angle among these individuals averaged at approximately 23.4° (±7.83°).

The ATR averaged at approximately 5.36° (±2.35°). A functional leg length discrepancy (FLLD) was observed in approximately 40% of the patients. Approximately 25% of cases involved the right limb, while approximately 15% involved the left limb.

Patients followed up for an average of approximately 28.1 (±14.5) months, with at least half of them having follow-ups of no more than 18 months.

Before initiating therapy, one-third of the children were classified into the HRS group, and two-thirds into the MRS group (regardless of age group). The most significant effects of therapy, as evidenced by the reduction in children in the HRS group, were that the number of 6–9-year-old group members declined by 22.4%, followed by a decrease of 10.6% in the 13–16-year-old group, and the smallest decrease (3.4%) in the 10–12-year-old group, which may be attributed to the period of accelerated growth in this age group (Table 2).

Among the participants, only 11.5% of children required observation after therapy. During the study period, approximately 6% of children withdrew from treatment due to financial difficulties, school obligations, or a non-acceptance of the treatment regimen. The vast majority (99.2%) of children who underwent a course of treatment did not require referral for orthopedic surgery. Only a few individuals from the group with highly advanced scoliosis were consulted at orthopedic clinics, of which two underwent scoliosis surgery. All decisions to terminate treatment, particularly for the group treated with a brace, were based on SOSORT recommendations [53].

The McNemar test, which evaluates the movement of patients between risk groups at distinct time points, demonstrated the statistical significance of the efficacy of therapy for the entire cohort as well as in individual age groups (Table 3).

Statistically significant reductions in ATR value change before and after the intervention were observed within each spinal section (*p*-value < 0.001) (Table 4).

A significant change in the Cobb angle was observed only in the Th-L section, with an average difference of 2.55° (*p* = 0.0061) (Table 5).

The Cobb angle measured during X-ray examination improved in 44.7% of cases for the thoracic section, 61.1% for the thoracolumbar section, and 44.2% for the lumbar section. A worsening of this angle was observed in 17.3%, 8.9%, and 21.8% of patients for each section, respectively.

In the case of the ATR angle, a greater percentage of improvement was noted, with 51.7% of patients improved for the thoracic section, 64.6% for the thoracolumbar section, and 56.9% for the lumbar section. However, the condition deteriorated in 23.6%, 16.2%, and 16.5% of patients for each section, respectively.

In cases of FLLD, length compensation was observed in 33% of children with asymmetry/scoliosis in the thoracic section, 41% in the thoracolumbar section, and 37% in the lumbar section. The occurrence of FLLDs after therapy was observed only in an average of 1.3% of cases (Table 6).

It is also worth noting that the therapy model used influenced the compensation of shortening regardless of the shortened side. The proportion test showed no significant differences between the proportions (*p* < 0.001) in the three analyzed spinal locations, indicating similar effectiveness of the method in all tested scoliosis locations (Table 7).

## 4. Discussion

The present study contributes to a growing trend among researchers to broaden postural assessment components beyond ATR measurement, reflecting a more patient-centered approach that incorporates function and quality of life [4,52].

The aspect considered in the early use of PSSE is closely related to estimating the risk of progression, which thus far relies on relatively small (*n* < 1000) epidemiological studies analyzing the extent of scoliosis on X-rays [54,55,56,57,58].

Using parameters such as the Cobb angle, rotation angle, Risser scale, and the child’s age and gender, we can assess the risk of scoliosis progression [56], and subsequently utilize the SOSORT guidelines [4] to determine therapeutic procedures. The presented study underscores that identifying FCSP in a child can be valuable in predicting the onset of idiopathic scoliosis (IS) solely through clinical assessment. It suggests qualifying children for early PSSEs with a lower ATR threshold of 5° if at least one FCSP is present. In addition to analyzing parameters determining the magnitude of scoliosis, this study examined one of the factors regarded as an FCSP, namely FLLDs.

Post-treatment, there was a notable migration of children from the HRS and MRS groups to the LRS group, observed in a total of *n* = 93 (11.5%) cases.

The most significant effectiveness of early PSSEs was observed in the 6–9-year-old group, with a reduction in the number of children in the HRS group by 22.4%, while the lowest effectiveness was noted in the 10–12-year-old group, with a reduction of only 3.4%. This difference may be attributed to the occurrence of pubertal growth spurts typically experienced by girls in this age group in the country where the study was conducted.

The analysis of children’s migration between IS risk groups using the McNemar test (Table 3) revealed a statistically significant impact of early PSSEs in each age group individually and in the entire cohort.

Following treatment, there was a significant decrease in the mean ATR across all three locations of scoliosis. However, a significant reduction in the Cobb angle was observed only in the thoracolumbar section, with a decrease of 2.55°; *p* < 0.05. It is noteworthy that a reduction in the mean Cobb angle was also observed in other spine segments. Comparing these findings with the literature, similar results were presented by Negrini et al. [59] in a long-term follow-up period (58 months) using the PSSE SEAS method, showing decreases in the mean Cobb angle in the thoracic (3.89 ± 6.26°), thoracolumbar (6.67 ± 6.79°), and lumbar spine (7.41 ± 7.22°). Considering that, in our study, the average Cobb angle before treatment was 23.4 ± 7.83°, and the risk of IS progression in the study group ranged from 40% to 65% depending on age according to Lonstein and Corlson [55], the treatment outcome appears highly positive. Furthermore, it is noteworthy that surgical intervention was only required for two individuals in the study group, which demonstrates better outcomes compared to previous reports [8,52,60,61,62,63].

The observed outcome could potentially be attributed to the early implementation of PSSEs, the comprehensiveness of the therapy model, and the long-term close cooperation of the therapeutic team. Changes in the Cobb angle were analyzed in children based on the location of scoliosis, and they were classified as improvement, stabilization, or deterioration according to SOSORT criteria [52]. Optimal results were noted in the thoracolumbar section, where stabilization or improvement was observed in 91.1% of children, while the least favorable outcomes were observed in the lumbar section (78%). Similar findings were reported by Negrini et al. [63], who also achieved the best treatment outcomes for thoracolumbar scoliosis. This may be attributed to the favorable distribution of curvature-correcting forces exerted by the brace [64].

In a study by Kwan et al. [65], which evaluated the PSSE Schrott method combined with bracing over a similar observation period (18 ± 6.2 months) and in a group with a comparable mean age (12.3 ± 1.4 years), similar results were obtained: 17% of patients experienced a reduction in the Cobb angle, stabilization was observed in 62%, and progression in 21%. On the other hand, in a randomized controlled trial assessing the effectiveness of the PSSE SEAS method, Monticone et al. [66] achieved slightly better results, with progression observed in only 8% of the subjects.

Considering the changes in FLLDs observed in the presented study, it is notable to mention the high effectiveness of early PSSEs in compensating for functional shortening, as the occurrence percentage in the entire population decreased from 40% to 1.1–1.4%.

Numerous studies emphasize the impact of functional leg length discrepancy (FLLD) in childhood on the development of internal stresses within pelvic structures, potentially leading to structural adaptive changes and asymmetry within the pelvis. This, in turn, can contribute to disturbances in spinal development [34,67,68,69,70].

Studies by Moseley [35] and D’Amico [41] have indicated that equalizing FLLDs during the treatment of idiopathic scoliosis (IS), as well as posterior superior iliac spine (PSIS) planes, is a desirable outcome.

While researchers generally acknowledge the existence of FLLDs, there remains a lack of consensus regarding the degree of FLLDs considered clinically significant, particularly in children [12,27,28,57]. The method of assessing FLLDs adopted in this study, which involved comparing heel levels in a supine position along with a criterion of 0.5 cm, may therefore be considered an interesting proposition.

Grivas et al. [17] suggest that FLLDs affect 3–15% of the population and may result from muscle contractures, biomechanical issues in pelvic joints, and dysfunction in other lower limb joints. Consequently, the early detection of FLLDs may not only benefit IS treatment but also aid in correcting lower limb deformities. This assertion is supported by research on the etiopathogenesis of orthopedic conditions in both children and adults [67,71,72,73,74,75,76].

Advancements in understanding the mechanisms of postural control and spine stabilization have opened up opportunities for correcting control deficits through the functional plasticity of the nervous and musculoskeletal systems. Research suggests that the body schema is typically a stable yet adaptable representation within the central nervous system, capable of being updated through sensory experiences [29]. Children with idiopathic scoliosis (IS) may lack a clear perception of trunk misalignment, leading their body schema to gradually adapt to the scoliotic state without a conscious awareness of the deformity [77,78,79].

The implementation of early PSSEs in children with IS aligns with the multifaceted objectives of conservative treatment outlined by the Society on Scoliosis Orthopedic and Rehabilitation Treatment (SOSORT). These objectives encompass not only enhancing spinal morphology and function but also improving overall well-being and preventing secondary overload and degenerative changes in the musculoskeletal system, which could adversely affect mental health and diminish adult quality of life [41].

Limitations of this study include potential inconsistencies in the type and duration of exercises performed by children at home under parental supervision. Additionally, the study’s single-center design and absence of a moderate-risk scoliosis (MRS) control group are notable limitations. Future research endeavors should involve larger sample sizes and incorporate control groups to yield more comprehensive evidence regarding the effectiveness of this therapeutic approach.

## 5. Conclusions

The early implementation of specific physiotherapy may enhance the efficacy of idiopathic scoliosis treatment by attenuating factors contributing to its progression.

## Figures and Tables

**Table 1:** Patient characteristics.

Variable	Parameter	Total (*n* = 812)
Study group	*n*	812
	Mean age (SD)	10.66 (3.16)
	Median (Q1–Q3)	10 (8–13)
	Range	6–16
Age division [years]	6–9	27% (*n* = 219)
	10–12	32.3% (*n* = 262)
	13–16	40.8% (*n* = 331)
Gender	Girls	65.5% (*n* = 532)
	Boys	34.5% (*n* = 280)
Risk groups for IS	Medium (ATR 5–6° or Hum Sump ≤ 7°)	66.3% (*n* = 538)
	High (ATR ≥ 7° or Hum Sump ≥ 8)	33.7% (*n* = 274)
X-ray patients from the HRS	33.7% (*n* = 274).	
	Only one X-ray	11.4% (*n* = 93)
	More than one X-ray 22.3% (*n* = 181)	
	Mean cobb angle (SD)	23.4° (7.83°)
	Median (Q1–Q3)	21° (15–30°)
	Range	10–50°
ATR angle	*n*	1113
	Mean ATR (SD)	5.36° (2.35°)
	Median (Q1–Q3)	5° (4–6°)
	Range	3–25°
Functional shortening of the lower limb	Left	15.4% (*n* = 125)
	Right	25.4% (*n* = 206)
	Equal	59.2% (*n* = 481)
Duration of treatment and observation [months]	*n*	812
	Mean (SD)	28.1 (14.5)
	Median (Q1–Q3)	18 (9–40)
	Range	1–67

**Table 2:** The summary of the changes in the number of patients in risk groups before and after therapy.

Age (Sample Size)	Time Point	Risk Groups for IS		Patients Referred for Observation
		High: ATR ≥ 7° or Hum Sump ≥ 8°	Medium: ATR 5–6° or Hum Sump ≤ 7°	
6–9 years old (*n* = 219)	Before therapy	33.8% (*n* = 74)	66.2% (*n* = 145)	
	After therapy	11.4% (*n* = 25)	66.2% (*n* = 145)	22.4% (*n* = 49)
10–12 years old (*n* = 262)	Before therapy	30.9% (*n* = 81)	69.1% (*n* = 181)	
	After therapy	27.6% (*n* = 73)	69% (*n*= 180)	3.4% (*n* = 9)
13–16 years old (*n* = 331)	Before therapy	36% (*n* = 119)	64% (*n* = 177)	
	After therapy	25.4% (*n* = 84)	64% (*n* = 212)	10.6% (*n* = 35)
Total *n* = 812	Before therapy	33.7% (*n* = 274)	66.3% (*n* = 538)	
	After therapy	22.3% (*n* = 181)	66.6% (*n* = 539)	11.5% (*n* = 93)

**Table 3:** The McNemar test for risk groups at two time points: before and after therapy.

Age	Time Points		Before Therapy		*p*-Value
			MRS	HRS	
6–9 years (*n* = 219)	After therapy	MRS	66.2% (*n* = 145)	22.4% (*n* = 49)	<0.001
		HRS	0% (*n* = 0)	11.4% (*n* = 25)	
10–12 (*n* = 262)		MRS	69% (*n* = 180)	3.4% (*n* = 9)	0.0133
		HRS	0% (*n* = 0)	27.6% (*n* = 73)	
13–16 (*n* = 331)		MRS	64% (*n* = 212)	10.6% (*n* = 35)	<0.001
		HRS	0% (*n* = 0)	25.4% (*n* = 84)	
Total (*n* = 812)		MRS	66.3% (*n* = 538)	11.5% (*n* = 92)	<0.001
		HRS	0% (*n* = 0)	22.3% (*n* = 181)	

**Table 4:** Change in ATR before and after treatment in relation to three locations of asymmetry/scoliosis and the results of the Wilcoxon test.

	Parameter ATR Angle	Before Treatment	After Treatment	*p*-Value
Thoracic section (Th)	*n*	352	352	<0.001
	Mean (SD)	5.42 (2.78)	4.83 (2.94)	
	Median (Q1–Q3)	5 (4–5)	4 (3–5)	
	Range	3–25	0–23	
Thoracolumbar section (Th-L)	*n*	396	396	<0.001
	Mean (SD)	5.29 (1.91)	4.07 (1.93)	
	Median (Q1–Q3)	5 (4–6)	4 (3–5)	
	Range	3–16	0–14	
Lumbar section (L)	*n*	267	267	<0.001
	Mean (SD)	5.36 (2.08)	4.52 (2.59)	
	Median (Q1–Q3)	5 (4–6)	4 (3–5)	
	Range	3–14	0–12	

**Table 5:** Changes in Cobb angle on X-ray before and after treatment in relation to three locations of scoliosis and the results of the Wilcoxon test.

	Parameter: Cobb Angle	Before Treatment	After Treatment	*p*-Value
Thoracic section (Th)	*n*	75	75	0.2543
	Mean (SD)	24.53 (12.33)	23.49 (13.02)	
	Median (Q1–Q3)	22 (15.5–30)	21 (14.5–30)	
	Range	10–50	5–66	
Thoracolumbar section (Th-L)	*n*	37	37	0.0061
	Mean (SD)	21.49 (7.86)	18.94 (8.23)	
	Median (Q1–Q3)	21 (15–25)	18 (12–24)	
	Range	10–45	8–41	
Lumbar section (L)	*n*	69	69	0.7505
	Mean (SD)	23.35 (11.51)	23.17 (12.31)	
	Median (Q1–Q3)	20 (15–30)	23 (14–32)	
	Range	10–50	5–63	

**Table 6:** Changes in ATR and Cobb angle on X-ray before and after treatment in relation to the location of asymmetry/scoliosis.

Parameter and Sample Size	Results after Treatment	Thoracic Section (Th)	Thoracolumbar Section (Th-L)	Lumbar Section (L)
X-ray (Cobb angle) *n* = 181		*n* = 75	*n* = 37	*n* = 69
	stabilization	38% (*n* = 69)	30% (*n* = 54)	34% (*n* = 62)
	worsened	17.3% (*n* = 31)	8.9% (*n* = 16)	21.8% (*n* = 39)
	improvement	44.7% (*n* = 81)	61.1% (*n* = 111)	44.2% (*n* = 80)
ATR angle *n* = 812		*n* = 352	*n* = 396	*n* = 267
	stabilization	24.7% (*n* = 87)	19.2% (*n* = 76)	26.6% (*n* = 71)
	worsened	23.6% (*n* = 83)	16.2% (*n* = 64)	16.5% (*n* = 44)
	improvement	51.7% (*n* = 182)	64.6% (*n* = 256)	56.9% (*n* = 152)
Change in FLLDs after treatment *n* = 812		*n* = 352	*n* = 396	*n* = 267
	FLLD = 0 before and after treatment	65.3% (*n* = 230)	57.3% (*n* = 227)	61.8% (*n* = 165)
	Left	1.4% (*n* = 5)	1.3% (*n* = 5)	1.1% (*n* = 3)
	It withdrew after treatment	33.2% (*n* = 117)	41.4% (*n* = 164)	37.1% (*n* = 99)

**Table 7:** Changes in the FLLDs of patients before and after treatment, with the results of Fisher’s proportion test.

	Shortening side	Shortening of the lower limb before treatment			*p*-value
		Thoracic (Th), *n* = 352			
		Left: 13.6%, *n* = 48	Equal: 64.6%, *n* = 227	Right: 21.8%, *n* = 77	<0.001
Shortening of the lower limb after treatment	Left (*n* = 8)	2%, *n* = 7	0.3%, *n* = 1	0%, *n* = 0	
	Equal (*n* = 328)	11.6%, *n* = 41	63.6%, *n* = 224	17.9%, *n* = 63	
	Right (*n* = 16)	0%, *n* = 0	0.6%, *n* = 2	4%, *n* = 14	
		Thoracolumbar (Th-L), *n* = 396			<0.001
		Left: 15.2%, *n* = 60	Equal: 56.1%, *n* = 222	Right: 28.8%, *n* = 114	
	Left (*n* = 14)	3.5%, *n* = 14	0%, *n* = 0	0%, *n* = 0	
	Equal (*n* = 348)	11.6%, *n* = 46	55.3%, *n* = 219	21%, *n* = 83	
	Right (*n* = 34)	0%, *n* = 0	0.8%, *n* = 3	7.8%, *n* = 31	
		Lumbar (L) (*n* = 267)			<0.001
		Left: 18%, *n* = 48	Equal: 60.7%, *n* = 162	Right: 21.3%, *n* = 57	
	Left (*n* = 12)	4.1%, *n* = 11	0.4%, *n* = 1	0%, *n* = 0	
	Equal (*n* = 239)	13.9%, *n* = 37	60.3%, *n* = 161	15.4%, *n* = 41	
	Right (*n* = 16)	0%, *n* = 0	0%, *n* = 0	6%, *n* = 16	

## Data Availability

The datasets generated and/or analyzed in the current study are available from the corresponding author upon reasonable request.
